# A Prospective Study of Soft Tissue Tumors Histocytopathology Correlation

**DOI:** 10.1155/2014/678628

**Published:** 2014-04-28

**Authors:** Priyanka Bhatia Soni, Anand Kumar Verma, Raj Kumar Chandoke, Jitendra Singh Nigam

**Affiliations:** ^1^Department of Pathology, Deen Dayal Upadhyay Hospital, Hari Nagar, New Delhi 110066, India; ^2^Department of Pathology, Employees' State Insurance Scheme of India (ESI) Hospital, Basaidarapur, New Delhi 110015, India; ^3^Department of Pathology, Saraswathi Institute of Medical Sciences, Hapur, Uttar Pradesh 245304, India

## Abstract

*Background.* Soft tissue tumors are defined as nonepithelial extraskeletal tissue of the body exclusive of the reticuloendothelial system, glia, and supporting tissue of various parenchymal organs. The absence of recognizable tissue architectural patterns in cytological preparation makes diagnosis by FNAC more difficult. *Aims.* To assess the utility of FNAC in diagnosing soft tissue tumors and to determine their patterns compared with with the respective histopathology results. *Materials and Methods.* 150 cases of soft tissue tumors were included in this study for cytologic and histologic correlation. FNAC air dried smears were stained with Giemsa stain and 95% ethanol fixed smears were stained with Papanicolaou stain. The smears were studied for cytological diagnosis and were categorized as benign, suspicious of malignancy, and malignant along with specific subtyping of the lesion. All diagnostic FNAC results were compared for diagnostic concordance using histology results as the “gold standard.” *Results.* The sensitivity, specificity, positive predictive value, negative predictive value, and efficiency were 70%, 100%, 97.90%, 100%, and 98%, respectively. *P* value was <0.0001 which shows statistically extreme significant correlation. *Conclusion.* FNAC is a very important preliminary diagnostic tool in palpable soft tissue lumps with high degree of correlation with the final histopathology report.

## 1. Introduction

The field of soft tissue tumors (STT) is enormously vast, and yet as cytologically, relatively undiscovered. The rarity of primary tumors of soft tissue and large range of different types of tumors, the diagnosis and classification of soft tissue tumors become most difficult areas in surgical pathology and absence of recognizable tissue architectural patterns in cytological preparation makes diagnosis by fine needle aspiration cytology (FNAC) even more difficult [1]. STT are defined as mesenchymal proliferations that occur in the extraskeletal, nonepithelial tissues of the body, excluding the viscera, coverings of the brain, and lymphoreticular system, and benign tumors are more common than malignant counterparts (sarcomas) with a ratio of at least 100 : 1 [2]. FNAC is almost painless, easy to perform, safe, and cost effective, without any anesthesia, and acts as a useful diagnostic technique in the initial diagnosis of tumors [3]. FNAC is fairly specific and sensitive in the diagnoses of primary, recurrent, and metastatic STT [3]. For distinguishing benign from malignant soft tissue tumors FNAC was very useful except for exact categorization of tumors, where it was not so effective [4]. However FNAC as a preliminary diagnostic tool offers several advantages as it can provide a predictive diagnosis of a benign or malignant neoplasm. In benign neoplasm, surgery can be avoided in the patients who are of poor surgical risk, and in malignant or recurrent cancers, FNAC allows the administration of a palliative treatment [5]. Present study was performed with the aim of assessing the utility of FNAC in diagnosing STT and determining their patterns compared with the respective histopathology results.

## 2. Materials and Methods

The study was undertaken in the Department of Pathology, Employees' State Insurance Scheme of India (ESI) Hospital, Basaidarapur, New Delhi, from February 2009 to February 2011. A total of 150 cases of soft tissue tumors were included in this study for cytologic and histologic correlation. Patients with palpable soft tissue lumps of size more than 1 cm were included in this study. Complete clinical details, examination findings, and radiological investigations of all patients were studied. FNAC was done with 23/24G needle attached to 10 mL disposable plastic syringe and air dried smears were stained with Giemsa stain and 95% ethanol fixed smears were stained with Papanicolaou stain if required. The smears were studied for cytological details/diagnosis and were categorized as benign, suspicious of malignancy, and malignant along with specific subtyping of the lesion. The smears were also assessed for the principle pattern shown by the tumor cells. The excised tissue specimens of all the above cases were processed routinely and stained with hematoxylin and eosin and examined, while special stains/immunohistochemistry was performed as and when required. All diagnostic FNAC results from patients who underwent a subsequent surgical excision were compared for diagnostic concordance using histology results as the “gold standard.” In addition FNAC results were analyzed for ability to recognize malignancy using statistical parameters of sensitivity, specificity, positive predictive value, and negative predictive value. Efficacy of FNAC in the diagnosis of soft tissue tumors was determined by calculating efficiency.

## 3. Results

**Figure 1:**
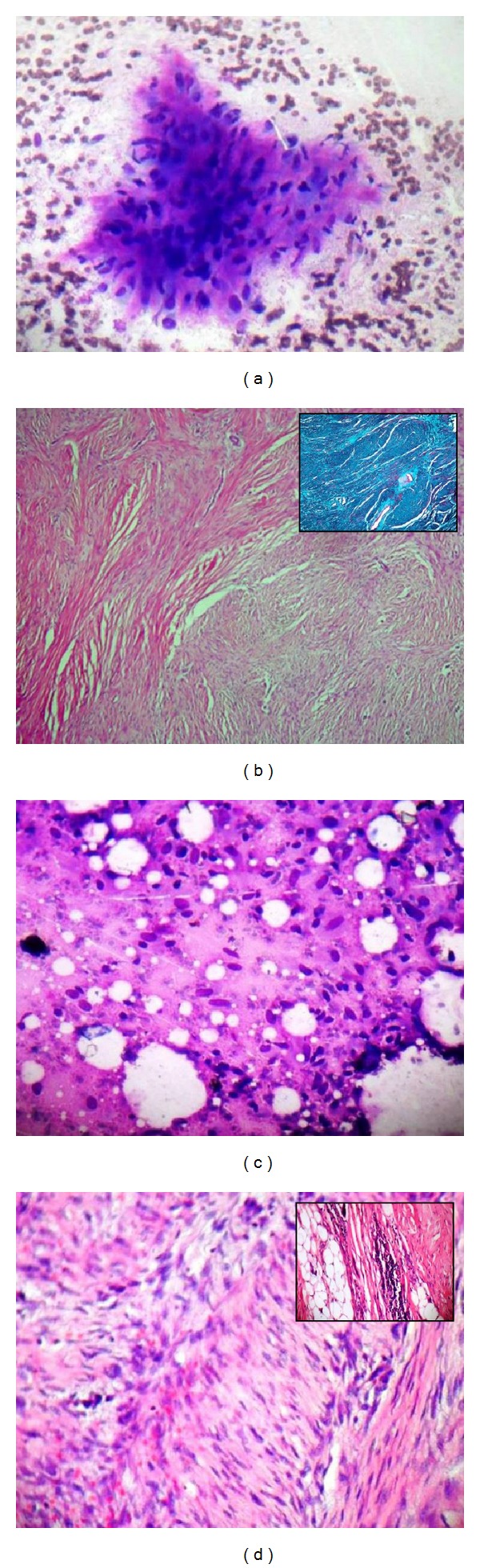
(a) Benign mesenchymal lesion (nodular fasciitis) cytology: myxoid background matrix with dispersed fibroblasts (Giemsa ×100). (b) Nodular fasciitis: plump, randomly oriented spindle shaped cells surrounded by myxoid stroma with dominating fibrous component (inset, Masson Trichrome) (H&E ×200). (c) Benign mesenchymal lesion (extra-abdominal desmoid) cytology: loosely arranged fibroblast-like cells with ovoid nuclei and greyish blue cytoplasm (Giemsa ×100). (d) Extra-abdominal desmoids: cellular proliferation of bland spindle shaped cells arranged into ill-defined long fascicles along with infiltration of spindle cells (inset) between skeletal muscle and fat (H&E ×100).

**Figure 2:**
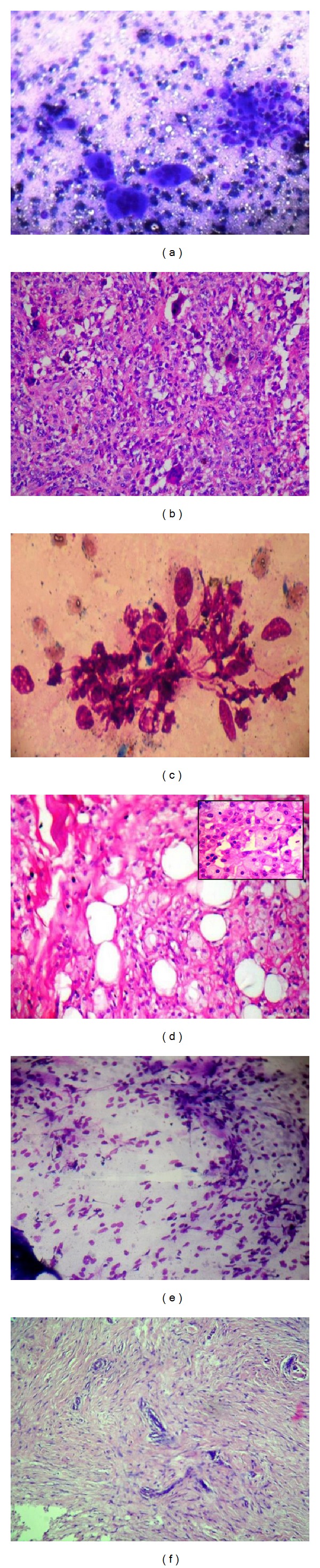
(a) Benign mesenchymal lesion (giant cell tumor of tendon sheath) cytology: dispersed rounded cells with rounded nuclei exhibiting moderate cellular pleomorphism and osteoclast like giant cells (Giemsa ×100). (b) Giant cell tumor of tendon sheath: mononuclear histiocytoid cells with variably prominent eosinophilic cytoplasm and scattered osteoclastic giant cells (H&E ×200). (c) Benign mesenchymal lesion (tuberous xanthoma) cytology: ovoid to plump benign spindle cell clusters (Giemsa ×400). (d) Tuberous xanthoma: large and small aggregates of foamy cells admixed with few nonfoamy cells and interspersed collagen bundles (H&E ×100, inset ×400). (e) Benign mesenchymal lesion (neurofibroma) cytology: highly cellular smears consisting of plump to tapered spindle shaped cells (Giemsa ×100). (f) Neurofibroma: arranged tumor cells nuclei which show a typical fascicular pattern of growth and serpentine shape (arrow) (H&E ×200).

On FNAC 95.3% (143/150) of patients had benign lesions, 3.34% (5/150) had malignant lesions, and 1.3% (2/150) were suspicious. The commonest age group for benign soft tissue lesions was between 2nd and 4th decades and for malignant lesions was between 4th and 5th decades of life. 54% (81/150) of cases with soft tissue tumors including both benign and malignant were males and 46% (69/150) were females. The commonest site of involvement of the benign tumors was upper extremities 43.5% (61/140) cases, followed by trunk and the head and neck region; however for malignant lesions it was the lower extremities followed by trunk region. On cytology the commonest benign lesion was lipoma in 48.2% (69/143) followed by vasoformative lesion (hemangioma) in 16% (23/143). All the benign lesions which could not be categorized into a particular group were labeled as BML (BML) 27.27% (39/143) (Figures 1 and 2).

The FNA of lipoma in all cases showed a uniform pattern consisting of fragments of adipose tissue composed of clusters of univacuolated fat cells and a small dark peripheral nucleus without evidence of atypia and confirmed on histology with few variants (Table 2). The FNAC of liposarcoma showed lipoblasts with significant mild to moderate pleomorphism, with focal areas showing adipocytic nuclear atypia, and the diagnosis of malignant adipocytic lesion was given.

The FNAC of nodular fasciitis cellular aspirates consisting of dispersed cells mixed with packed clusters of spindle shaped cells showing marked pleomorphism with fusiform nuclei, fine chromatin, and cytoplasmic processes. Few plump cells with ovoid, rounded, and irregular nuclei were also found, lying against myxoid background matrix. Two cases were accurately diagnosed on cytology.

FNAC of calcifying aponeurotic fibroma, collagenous fibroma, and fibroma of tendon sheath showed spindle cells lying dispersed and in packed clusters against clean background, showing predominantly a spindle cell pattern. None of them could be categorized broadly and were labelled as BML cytologically.

FNAC of extra-abdominal desmoids tumor showed good cell yield, with spindle cells lying singly and in loose clusters against a fatty and myxoid background. Few cells showed tapered and wavy nuclei. Cytological diagnosis of BML, probably of neurogenic origin, was given, thus not giving an accurate diagnosis. Diagnosis was given on histology and immunohistochemistry.

Keloid FNAC smears showed very few scattered spindle shaped cells with glassy collagenized material and were not correctly diagnosed and confirmed on histology.

FNAC of low grade fibromyxosarcoma yielded myxoid material and smears showed moderate cellularity of loosely cohesive spindled fibroblast-like cells with moderate atypia arranged in a myxoid background. However already bland looking cells were masked with abundant myxoid material and so the nuclear features were not too prominent to call them malignant and therefore diagnosis given was suspicious for malignancy.

FNAC of giant cell tumor of tendon sheath showed mild to moderate cellularity, composed of numerous dispersed and clustered mononuclear rounded cells showing moderate amount of pleomorphism ranging from plump stellate to round cells. In addition, multinucleated giant cells of histiocytic origin were also seen, the nuclei of which resembled those of stromal cells. In the background brown pigment laden macrophages and few inflammatory cells were observed. Accurate cytological diagnosis was rendered in all the cases and duly confirmed by subsequent histopathological study.

FNAC of benign fibrous histiocytoma showed solid cell clusters and dispersed spindle shaped cells, thereby giving predominantly a spindle cell smear pattern. However, in none of the cases accurate broad categorization could be done and thus a diagnosis of BML was given.

FNAC of dermatofibroma showed low cellularity consisting of scattered spindle shaped cells lying against a fatty background. It was labelled as BML and could not be broadly categorized.

FNAC of tuberous xanthoma yielded scant aspirate, with low cellular smears showing scattered spindle shaped cells. Accurate diagnosis could not be made and so labelled as BML.

FNAC of undifferentiated pleomorphic sarcoma yielded blood admixed aspirate and smears showed high cellularity composed of bizarre tumor cells lying singly and entangled clusters. Individual cells were round to oval with indistinct cytoplasm, high nuclear : cytoplasmic ratio (N : C ratio), and prominent nucleoli and labelled as malignant mesenchymal tumor (MMT).

FNAC of dermatofibrosarcoma protuberans cases showed scanty to moderate cellularity, consisting of small cohesive clusters of short spindle cells and a diagnosis of BML was given.

FNAC of angioleiomyoma showed poor cell yield with predominating dissociated spindle cells lying singly against bloody background in one case and slightly collagenous in another. Exact categorization could not be done and labelled as BML.

The FNAC of pericytic tumor shows uniform pattern consisting of fragments of adipose tissue composed of a single large vacuole of fat and a small dark peripheral nucleus with no evidence of atypia. A cytological diagnosis of lipoma was given, thus not accurately diagnosed.

FNAC of vascular tumors showed yielded copious blood. Majority of the smears were poor in cells and showed blood only. Few cases showed spindle shaped cells with ovoid nuclei and a varying amount of indistinct cytoplasm. All cases were diagnosed as benign vascular lesion except one case showing occasional cohesive epithelioid cells with moderate cytoplasm, ovoid nuclei, and small nucleoli with mildly pleomorphic nuclei and irregular nuclear contours against a largely bloody background and diagnosed as vascular BML.

FNAC of chondroosseous lesion showed spindle cells arranged in packed clusters only. Broad categorization could not be done and diagnosis given was BML.

FNAC of schwannoma showed moderate cellularity with mainly cohesive spindle cell clusters having long-slender nuclei with pointed ends and indistinct cytoplasm with at places the arrangement was suggestive of palisading. A probable diagnosis of neurilemmoma was given in 5 cases only as they experience sharp radiating pain when tumor was aspirated and this would also suggest a neurogenic tumor. The rest of the 10 cases were not broadly categorized and showed mainly spindle cell clusters and cells lying singly and were labelled as BML.

FNAC of four cases of neurofibroma showed good cellularity consisting of clustered and scattered spindle shaped, elongated cells with poorly preserved, pale staining cytoplasm and irregular, elongated nuclei. Scattered naked nuclei were also noted. These cases were accurately diagnosed on FNAC. Two cases of neurofibroma showed moderate to high cellularity consisting of occasional clustered aggregates of plump to tapered spindle cells with irregular, hyperchromatic nuclei. These were diagnosed as BML.

FNAC of traumatic neuroma yielded scant aspirate. Hypocellular smears showed scattered spindle shaped cells lying singly and in small clusters. A diagnosis of BML was given.

FNAC of malignant peripheral nerve sheath tumor with low malignant potential yielded blood admixed aspirate. Smears showed mainly tissue fragments, with few dispersed cells. Individual cells were ovoid to spindle shaped admixed with wavy cells. The case was labelled as BML probably of neurogenic origin.

FNAC of alveolar soft part sarcoma yielded blood admixed aspirate. Smears show clusters of spindle shaped to epithelioid cells (polygonal) entangled in stroma. Numerous scattered plump cells were seen against dense hemorrhagic background. Individual cells show high N : C ratio, with round to oval irregular nucleus, prominent pale blue nucleoli, and scant cytoplasm. A diagnosis of malignant mesenchymal tumor with predominantly epithelioid smear pattern was given.

FNAC of PEComa yielded blood admixed material. Cellular smears show numerous scattered large round to oval spindle shaped cells with high N : C ratio and bizarre looking nuclei with prominent nucleoli. Few cells entangled in stroma were seen as well. Diagnosis made was malignant mesenchymal tumor, as it could not be accurately categorized.

FNAC of clear cell sarcoma showed loosely cohesive atypical spindle to oval cells entangled in stroma. Individual cells had high N : C ratio. Diagnosis given was malignant mesenchymal lesion.

**Figure 3:**
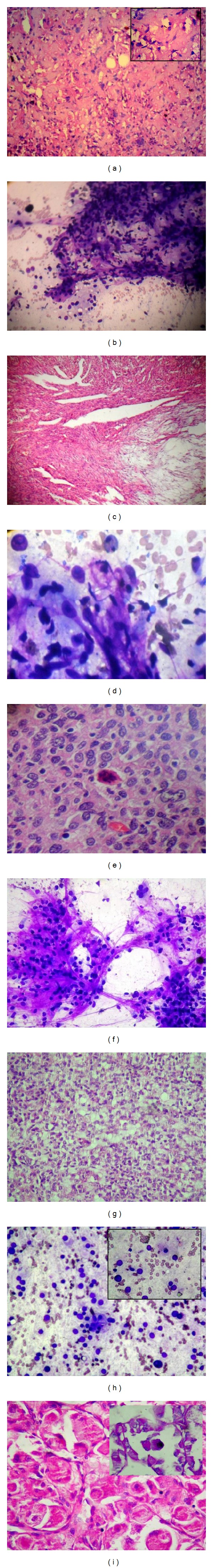
(a) Well differentiated liposarcoma: lipoblasts with atypical hyperchromatic nuclei and multinucleated giant cell (H&E ×100, ×400 inset). (b) Malignant mesenchymal lesion (low grade fibromyxoid sarcoma) cytology: loosely arranged bland spindle cells with slight to moderate atypia arranged in a myxoid background (Giemsa ×100). (c) Low grade fibromyxoid sarcoma: abrupt transition from fibrous areas (composed of bland spindle cells) to myxoid areas (H&E ×100). (d) Malignant mesenchymal lesion (malignant fibrous histiocytoma) cytology: bizarre tumor cells with prominent nucleoli (Giemsa ×400). (e) Malignant fibrous histiocytoma: cells with marked pleomorphism and mitosis (H&E ×400). (f) Malignant mesenchymal lesion (clear cell sarcoma) cytology: atypical spindle to oval cells entangled in stroma. (Giemsa × 400). (g) Clear cell sarcoma: Tumor cells in short fascicles separated by dense fibrous septa exhibiting prominent vesicular nuclei with large single nucleolus (H&E ×400). (h) Malignant mesenchymal lesion (alveolar soft part sarcoma) cytology: spindle to epithelioid cells with prominent nucleoli and high N : C ratio (Giemsa ×100, inset ×400). (i) Alveolar soft part sarcoma: tumor cells in typical organoid/nesting pattern are separated by delicate partitions of connective tissue containing vascular channels (H&E ×100) with chunky crystalline inclusions (inset) (PAS × 400).

All malignant lesions on cytology were labelled as malignant mesenchymal tumor (MMT) (Figure 3). Excluding lipoma, the most common pattern observed was spindle cell pattern in 48% (72/150), followed by myxoid pattern in 4% (6/150) (Table 1). On histopathological examination (Table 2) 93.33% (140/150) of the soft tissue tumors were benign and 6.67% (10/150) malignant. Amongst malignant lesions, two cases of liposarcoma, 2 cases of dermatofibrosarcoma protuberans, and one case of each of malignant peripheral nerve sheath tumor, low grade fibromyxosarcoma, undifferentiated pleomorphic sarcoma, alveolar soft part sarcoma, PEComa, and clear cell sarcoma were seen. Three cases reported as BML on cytology were diagnosed as dermatofibrosarcoma protuberans in two cases and malignant peripheral nerve sheath tumor in one case on histopathology. Three cases reported as MML on cytology were diagnosed as alveolar soft part sarcoma, undifferentiated pleomorphic sarcoma, and clear cell sarcoma on histopathology. Both the cases reported as suspicious for malignancy were malignant on histopathology (low grade fibromyxosarcoma and PEComa). One case of lipoma on FNA turned to be glomus tumor on histopathology.

Correlation of cytodiagnosis and histopathological diagnosis in case of benign soft tissue tumors showed accurate categorization in 77.14% of cases (108/140). (Table 3) Among individual tumors entity adipocytic tumor had the best cytohistological correlation with 100% (68/68) correctly identified on FNAC, followed by 95.84% correlation for vascular tumors (23/24). Least correlation was observed in cases of fibroblastic tumors 20% (2/10) followed by peripheral nerve sheath tumor 39.13% (9/23). 22.86% (32/140) of cases were diagnosed as benign but not accurately categorized. The correlation of cytodiagnosis and histopathological diagnosis in cases of malignant soft tissue tumors showed accurate categorization in only 2 cases (20%) out of 10 cases (Table 4). Three (30%) cases diagnosed as malignant on FNAC could not be accurately categorized. Two cytological suspicious diagnoses were evaluated histologically and both of them confirmed as malignancy whereas three cases diagnosed as benign on FNAC turned out to be malignant. Overall accurate/exact categorizations for both benign and malignant tumors (total STT studied) were seen in 70% (105/150) of cases. Because FNAC is a screening test with relatively high sensitivity for the diagnosis of malignancy, suspicious cases were included in the category of malignant lesions for statistical analysis. The sensitivity, specificity, positive predictive value, negative predictive value, and diagnostic accuracy of FNAC were calculated taking histology as gold standard. The sensitivity, specificity, positive predictive value, negative predictive value, and efficiency were 70%, 100%, 97.90%, 100%, and 98%, respectively. Pearson chi-square test with Yates' corrections was 87.665 with 1 degree of freedom and *P* value was <0.0001 which shows statistically extreme significant correlation.

## 4. Discussion

FNAC has numerous advantages over open biopsy, because it is relatively inexpensive and easy to perform and yields clinically useful results with a rapid turnaround time. Despite these benefits the present study depicts the difficulty of categorizing soft tissue lesion cytologically. If FNAC is to be of value the cytologists must be able to differentiate primary malignant soft tissue tumors from benign lesions and from other malignancies, such as metastasis of carcinomas and malignant lymphomas and tumors of dermal appendages. Kilpatrick et al. observed the extent to which cytology can be utilized in effective diagnosis of STT and influence on initial therapy in adults and pediatric sarcoma [6]. Rekhi et al. concluded that FNAC is fairly specific and sensitive in STT diagnoses for primary, recurrent, and metastatic lesions and the cytological types, especially round cell and pleomorphic sarcomas, can be quickly identified [3].

Parajuli and Lakhey also conclude that FNAC is highly sensitive to detect benign soft tissue tumors and highly specific for malignant soft tissue tumors [7]. Kulkarni et al. concluded that FNAC of STT provided acceptable diagnostic accuracy when supported by appropriate clinical and other diagnostic data [8]. The benign tumors are more common than malignant tumors with a ratio of at least 100 : 1 [2], and in the present study, ratio was 14 : 1. Roy et al. observed that benign STT were relatively common above third decade of life, while malignant STT occurred in patients of all ages [5]. Rekhi et al. observed that the commonest age group is 21–30 years for STT [3]. In present study, the commonest age group was 21–30 years and 40–50 years for benign and malignant STT, respectively. In present study, the commonest site for benign and malignant STT was upper extremities followed by trunk and the lower extremities followed by trunk region, respectively. Roy et al. observed that benign tumors are roughly equally distributed across all parts of the body with a slight predilection for the upper parts and the commonest site of involvement of the malignant tumors was trunk [5], in contrast to present study. Roy et al. [5] also observed that the most common benign tumour was lipoma followed by neurofibroma followed by haemangioma, and Parajuli and Lakhey [7] observed lipoma followed by benign mesenchymal tumor followed by benign fibrohistiocytic tumor, while in present study adipocytic tumours were commonest followed by vascular tumours followed by peripheral nerve sheath tumors. In malignant cases, Roy et al. observed malignant fibrous histiocytoma followed by rhabdomyosarcoma and liposarcoma [5]. In the present study tumors of uncertain differentiation were the commonest tumors followed by fibrohistiocytic and adipocytic tumors. The sensitivity, specificity, positive predictive value, negative predictive value, and efficiency in the present study were 70%, 100%, 97.90%, 100%, and 98%, respectively. Rekhi et al. observed the diagnostic accuracy of FNAC in STT was 98%, with a positive predictive value of 98% in malignant cases and a negative predictive value of 100% in benign cases [3]. Dey et al. observed the sensitivity, specificity, and positive predictive value of FNAC in diagnosis of STT were 91.5%, 92.5%, and 95.5%, respectively [4]. Roy et al. observed that the diagnostic accuracy of FNAC of STT for benign and malignant tumours was 90.6% and 91.3%, respectively, with overall accuracy rate of 90.8% [5]. In the present study overall accuracy rate was 98%. Parajuli and Lakhey observed that the overall diagnostic accuracy of FNAC in STT was 80% and the sensitivity and specificity of diagnosing benign soft tissue tumors were 97.36% and 66.67%, respectively, and for malignant soft tissue tumors 66.67% and 97.36%, respectively [7]. Kulkarni et al. observed that the overall diagnostic accuracy of FNAC in STT was 93.33% for all lesions, 93.93% for malignant mesenchymal tumour, 93.33% for benign mesenchymal lesion, and 100% for metastasic tumours [8]. Layfield et al. observed that the diagnostic sensitivity of FNAC for detecting malignant neoplasm was 95% and the specificity was 95% [9]. Wakely and Kneisl observed 100% sensitivity and 97% specificity in STT diagnosis with FNAC and concluded that aspiration cytopathology of soft tissue mass lesions using FNA biopsy can be an accurate and minimally invasive method for the initial pathologic diagnosis of primary benign and malignant soft tissue masses, for the pathologic confirmation of metastatic tumors to soft tissue and for the documentation of locally recurrent soft tissue neoplasms [10].

## 5. Conclusion

FNAC is a very important preliminary diagnostic tool in palpable soft tissue lumps and done by expert hands; the results show a high degree of correlation with the final histopathology report.

## Figures and Tables

**Table:** (a) Cytomorphological pattern of STT on FNAC.

S. no.	Pattern	Benign	Suspicious	Malignant	Total
1	Spindle cell	45	01	0	72
2	Pleomorphic	0	0	02	02
3	Myxoid	04	01	01	06
4	Small round cell	0	0	0	0
5	Epithelioid	0	01	0	01

**Table:** (b) Spectrum of soft tissue tumors on FNAC.

S. no.	Category	Benign	Malignant
1	Adipocytic tumor	Lipoma = 69	Atypical lipomatous tumor = 2
2	Fibroblastic tumors	Nodular fasciitis = 2	0
3	Fibrohistiocytic tumors	GCT = 06	0
4	Vascular tumor	23	0
5	Peripheral nerve sheath tumor	Neurofibroma = 04	0
6	Mesenchymal lesion	BML = 39	MML = 03

Total		143	05

Suspicious cases = 2.

**Table 2:** Spectrum of soft tissue tumors on histopathology.

Categories	Number of cases	Percentage (%)
Benign		
Adipocytic tumors	**68**	**45.34**
Classic lipoma	54	36
Angiolipoma	6	4
Myxolipoma	1	0.67
Fibrolipoma	6	1
Pleomorphic lipoma	1	0.67
Vascular tumors	**24**	**16**
Hemangioma	23	15.33
Epithelioid hemangioma	1	0.67
Peripheral nerve sheath and related tumors	**23**	**15.33**
Schwannoma	15	10
Neurofibroma	06	4
Traumatic neuroma	02	1.33
Fibrohistiocytic tumors	**11**	**7.34**
Giant cell tumor of tendon sheath	6	4
Benign fibrous histiocytoma	3	2
Dermatofibroma	1	0.67
Tuberous xanthoma	1	0.67
Smooth muscle tumors	2	1.33
Angioleiomyoma		
Pericytic tumors	1	0.67
Glomus tumor		
Fibroblastic tumors	**10**	**6.67**
Nodular fasciitis	3	2
Collagenous fibroma	1	0.67
Fibroma of tendon sheath	2	1.33
Calcifying aponeurotic fibroma	1	0.67
Extra abdominal desmoids	1	0.67
Keloid	2	1.33
Chondroosseous tumor	1	0.67
Soft tissue tumor		

Malignant		
Fibrohistiocytic tumors	3	2
Dermatofibrosarcoma protuberans	2	1.33
Tumors of uncertain differentiation	**3**	**2**
Alveolar soft part sarcoma	1	0.67
PEComa	1	0.67
Clear cell sarcoma	1	0.67
Undifferentiated sarcomas		
Undifferentiated pleomorphic sarcoma	1	0.67
Peripheral nerve sheath and related tumor	1	0.67
Fibroblastic tumors	1	0.67
Low grade fibromyxoid sarcoma		
Adipocytic tumors	2	1.33
Liposarcoma		

**Table 3:** Correlation of cytodiagnosis and histopathological diagnosis in benign tumors.

Histopathology	Total number of cases	Correlation with cytological diagnosis	
		Accurately categorized	Diagnosed as benign but not accurately categorized
Adipocytic tumors	68	68	0
Fibroblastic tumors	10	2	8
Fibrohistiocytic tumors	11	6	5
Smooth muscle tumors	2	0	2
Pericytic tumors	1	0	1
Vascular tumors	24	23	1
Chondroosseous tumors	1	0	1
Peripheral nerve sheath and related tumors	23	4	19

Total	140	108 (77.14%)	32 (22.86%)

**Table 4:** Correlation of cytodiagnosis and histopathological diagnosis in malignant tumors.

Histopathology	Total number of cases	Correlation with cytological diagnosis			
		Accurately categorized	Diagnosed as malignant but not accurately categorized	Diagnosed as benign on FNAC	Suspicious
Adipocytic tumors	2	2	0	0	0
Fibroblastic tumors	1	0	0	0	1
Fibrohistiocytic tumors	2	0	0	2	0
Peripheral nerve sheath and related tumors	1	0	0	1	0
Tumors of uncertain differentiation	3	0	2	0	1
Undifferentiated sarcomas	1	0	1	0	0

Total	10	2 (20%)	3 (30%)	3 (30%)	2 (20%)
